# Peri-Implant Health and Diagnostic Considerations

**DOI:** 10.3390/ijerph191912008

**Published:** 2022-09-22

**Authors:** Leonardo Mancini

**Affiliations:** Department of Life, Health and Environmental Sciences, University of L’Aquila, 67100 L’Aquila, Italy; leonardo.mancini@graduate.univaq.it

In recent years, implant rehabilitation has become a popular method for replacing lost teeth. Several studies have reported that dental implants can survive over 20 years when supporting diverse dental prostheses [1,2]. However, technical or biological complications may occur; thus, implant survival should be differentiated from implant success. The term “success” refers to the implant’s appearance at the time of the examination, implying no infections, radiolucency, or mobility, whereas the term “survival” refers to implants still in place at the time of examination regardless of the health and condition of the surrounding tissue and prosthetic rehabilitation [3,4]. Technical and biological complications are the most common types reported in the literature when assessing implant success or survival.

Generally, technical complications include all adverse effects associated with the fixture, including implant fractures, screw loosening, and chipping [4]. Conversely, biological complications tend to be characterized by an inflammatory response of the peri-implant tissues that may lead to either peri-implant mucositis or peri-implantitis [5]. Interestingly, for the first time, these conditions were considered in the latest classification of periodontal disease, and according to the latest Workshop of Periodontology 2017. 

Peri-implant mucositis can be defined as an inflammatory condition of the soft tissue surrounding the implant with redness, swelling, bleeding, and absence of marginal bone loss, whereas peri-implantitis is considered as an inflammatory status followed by loss of the supporting hard tissue around the implant [6] (Figure 1).

The diagnosis is made by means of a clinical examination, X-rays, and supporting data.

Clinical parameters currently used in daily clinical practice and epidemiological studies are bleeding on probing (BoP), probing depth (PD), marginal bone loss (MBL), and plaque index (PI). These parameters must be assessed to distinguish between healthy, mucositis, or peri-implantitis conditions. 

Despite the efforts of the latest Workshop to classify the disease and suggest different parameters and thresholds, recent studies have revealed that the classification proposed may not be sensitive enough to detect an early form of peri-implantitis [7]. For example, it is worth noting that there are different forms of BOP evaluation: punctiform or profuse bleeding and bleeding after measuring the probing depth or bleeding after walking with the periodontal probe through the peri-implant sulcus [8]. As these different methods have not been standardized in the literature, there remain controversial and unclear guidelines that suggest a precise type of score.

An example is the numerous types of bleeding score indexes published. The gingival bleeding score (GBS), gingival bleeding index (GI), papillary bleeding index (PBI), periodontal pocket bleeding index (PBS), modified papillary bleeding index (MPBI), bleeding time index (BTI), and the modified sulcular bleeding index (mSBI) are just a few of the indexes suggested for the dichotomous (yes/no) bleeding evaluation.

One way to overcome the limitations of BOP would be the global adoption of a standardized and reliable index.

A recent systematic review established that, among 159 longitudinal studies on the prevention and management of peri-implant disease, PD and BoP were reported in 89% and 87% of the studies, respectively, whereas PI and MBL were reported in only 64% and 49%, respectively. PD assessment was mentioned in 142 studies and a metal periodontal probe was used in 60% of the studies whereas a pressure-sensitive probe (18%) or plastic probe (11%) were used less frequently. The prosthesis was removed for probing purposes in only 6% of the studies. BoP showed similar heterogeneity, with 93 studies reporting only bleeding on probing alone and 46 evaluating bleeding and suppuration on probing. This measure was recorded in a binary fashion in 68% of the studies; only 15% reported grading and the use of an ordinal scale. Examiner calibration was performed in 39% to 47% of the studies [9].

These results demonstrate great heterogeneity in diagnostic criteria and collection of data. Hence, the last consensus tried to provide guidelines for clinical practitioners and epidemiological studies. However, the clinical evaluation was not deeply addressed in terms of the type of parameter, method of assessment or calibration.

Overall, this editorial underlines the need to unify and clarify the use of standardized diagnostic tools and parameters worldwide in order to reduce heterogeneity among studies and facilitate the collection and analysis of data.

In a clinical environment the most important aspect is to categorize and classify our patients and implants; therefore, being a private practitioner means translating clinical data into daily practice to support decision-making for any type of intervention and help discuss treatment with patients during the initial appointment. 

On the other hand, researchers are struggling with the selection of reproducible indexes [10]. Therefore, there is an urgent need to standardize the way to diagnose our patients and deliver data accurately and consistently. Only in this way, we might move one step further in improving current patient care and global oral health.

## Figures and Tables

**Figure 1:**
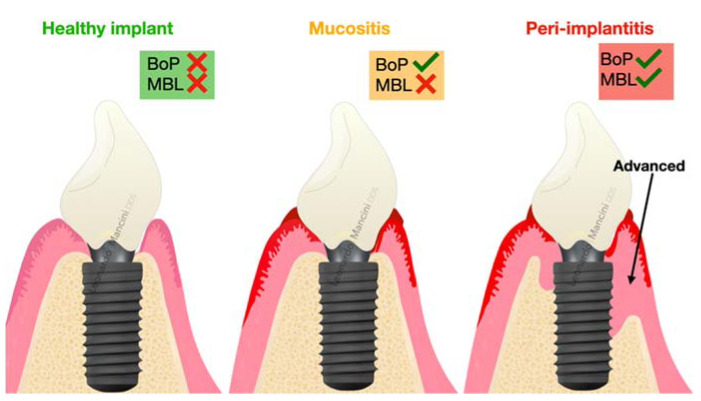
Illustration of the case definition for peri-implant health, mucositis and peri-implantitis in day-to-day clinical practice.
